# Das Basler Modell der prinzipienorientierten klinischen Ethikkonsultation 2.0: Eine Einführung für die Psychiatrie

**DOI:** 10.1007/s00115-024-01710-9

**Published:** 2024-07-20

**Authors:** Jan Schürmann, Charlotte Wetterauer, Anna Lisa Westermair, Manuel Trachsel

**Affiliations:** 1 Abteilung Klinische Ethik, Universitätsspital Basel (USB), Universitäre Psychiatrische Kliniken Basel (UPK), Universitäre Altersmedizin Felix Platter (UAFP), Universitäts-Kinderspital beider Basel (UKBB), Spitalstrasse 22, 4031 Basel, Schweiz; 2 Institut für Biomedizinische Ethik und Geschichte der Medizin, Universität Zürich, Zürich, Schweiz; 3 Medizinische Fakultät, Universität Basel, Basel, Schweiz

**Keywords:** Ethik, Klinische Ethikberatung, Indikation, Evidenz, Prinzipien der biomedizinischen Ethik, Ethics, Clinical ethics counselling, Indications, Evidence, Principles of biomedical ethics

## Abstract

**Hintergrund:**

Die Wirksamkeit klinischer Ethikberatung in der Medizin hinsichtlich der Zufriedenheit der Beteiligten, der Unterstützung ethischer Entscheidungsfindung, der wahrgenommenen Auswirkungen auf die klinische Versorgung, der moralischen Kompetenz und der Qualität der Kommunikation ist inzwischen empirisch gut belegt. Auch in der Psychiatrie verfügen immer mehr Einrichtungen über Strukturen klinischer Ethikberatung. Es fehlt jedoch nach wie vor an evaluativer Begleitforschung zum Nutzen und zur differenziellen Indikation der verschiedenen Formen und Modelle klinischer Ethikberatung in der Psychiatrie.

**Ziel der Arbeit:**

Der Artikel präsentiert die Grundlagen und die schrittweise Anwendung der prinzipienorientierten klinischen Ethikkonsultation nach dem Basler Modell 2.0.

**Material und Methoden:**

Der Artikel stützt sich auf Materialien und Verfahren, die an der Abteilung Klinische Ethik des Universitätsspitals Basel und der Universitären Psychiatrischen Kliniken Basel zur prinzipienorientierten klinischen Ethikkonsultation nach dem Basler Modell 2.0 entwickelt wurden.

**Ergebnisse und Diskussion:**

Die prinzipienorientierte klinische Ethikkonsultation nach dem Basler Modell 2.0 eignet sich, um moralische Fragen und Konflikte in der psychiatrischen Praxis zu bearbeiten und ethisch zu reflektieren. Es braucht jedoch weitere evaluative Begleitforschung zum Nutzen und zur differenziellen Indikation dieses und weiterer Modelle klinischer Ethikberatung in der Psychiatrie.

## Abstract (en)

**Background:**

The effectiveness of clinical ethics counselling in medicine in terms of satisfaction of the participants, support of ethical decision-making, perceived impact on clinical care, moral competence and quality of communication is now empirically well validated. In psychiatry, more and more institutions have structures for clinical ethics consultation as well; however, there is still a lack of evaluative accompanying research on the benefits and differential indications of the various forms and models of clinical ethics counselling in psychiatry.

**Aim of the work:**

The article presents the principles and the step by step application of the principle-oriented clinical ethics consultation according to the Basel model 2.0.

**Material and methods:**

The article is based on material and procedures developed at the Clinical Ethics Unit of the University Hospital Basel and the University Psychiatric Clinics Basel for the principle-oriented clinical ethics consultation according to the Basel model 2.0.

**Results and discussion:**

Principle-oriented clinical ethics consultation according to the Basel model 2.0 is suitable for dealing with moral issues and conflicts in psychiatric practice and for their ethical reflection; however, further evaluative accompanying research is needed on the benefits and differential indications of this and other models of clinical ethics support in psychiatry.

Die Wirksamkeit klinischer Ethikberatung in der Medizin in Bezug auf die Zufriedenheit der Beteiligten, die Unterstützung ethischer Entscheidungsfindung, die wahrgenommenen Auswirkungen auf die klinische Versorgung, die moralische Kompetenz und die Qualität der Kommunikation ist empirisch belegt. Auch in der Psychiatrie verfügen immer mehr Einrichtungen über Strukturen klinischer Ethikberatung. Die prinzipienorientierte klinische Ethikkonsultation nach dem Basler Modell 2.0 eignet sich, um moralische Fragen und Konflikte in der psychiatrischen Praxis zu bearbeiten.

## Klinische Ethikberatung in der Psychiatrie

### Ethische Fragen

Die *ethischen Herausforderungen*, mit denen sich psychiatrische Fachpersonen in ihrem Berufsalltag konfrontiert sehen, sind vielfältig [6, 11, 21]. So stellen sich erstens ethische Fragen auf der Ebene der einzelnen Patient*innen, etwa ob und unter welchen Umständen Interventionen gegen den Willen oder Widerstand von Patient*innen mittels Zwang gerechtfertigt sind oder wie die Behandlung von Personen mit schweren psychisch-somatischen Komorbiditäten gewährleistet werden kann. Zweitens stellen sich ethische Fragen in Situationen, in denen *Drittpersonen *betroffen sind, etwa ob vertrauliche Informationen zum Schutz Dritter weitergegeben werden dürfen oder wie Mitpatient*innen vor aggressivem Verhalten der Patient*in geschützt werden sollen. Drittens stellen sich ethische Fragen auf einer *systemischen Ebene*, etwa nach welchen Kriterien psychiatrische Leistungen verteilt bzw. behandlungsbedürftige Patient*innen triagiert werden sollen, wie in der forensischen Psychiatrie die therapeutischen Interessen von Patient*innen mit den Maßnahmen des Strafvollzugs in Einklang gebracht werden können oder ob Personen mit schweren, chronischen psychischen Erkrankungen ärztlich assistierten Suizid in Anspruch nehmen dürfen (zur Systematik der drei Typen ethischer Fragen vgl. [11]).

### Strukturen der klinischen Ethikberatung

Zur Unterstützung bei ethischen Fragen im klinischen Alltag etablieren sich in der Medizin zunehmend Strukturen der klinischen Ethikberatung. *Klinische Ethikberatung* ist eine strukturierte Beratung durch eine oder mehrere Ethikberater*innen, die Behandelnde, Patient*innen, Angehörige oder die Krankenhausadministration dabei unterstützt, Unsicherheit, Distress, Konflikte oder Dilemmata bezüglich ethischer Fragen in einem klinischen Fall bzw. Kontext aufzuklären, zu vermeiden oder zu lösen [1]. In den USA verfügen 86 % aller Krankenhäuser über eine Struktur zur Ethikberatung; 74 % bieten aktiv klinische Ethikberatung an [7]. Auch in europäischen Ländern wie Deutschland oder der Schweiz wird klinische Ethikberatung in Krankenhäusern zunehmend eingeführt [23, 28]. Um die Qualität der Ethikberatung zu verbessern, wurden Empfehlungen von Ärzteverbänden [29], Berufsstandards [25], Ausbildungsrichtlinien und -kurse [5] sowie in den USA Zertifizierungs- und Akkreditierungsprogramme und Evaluationsinstrumente entwickelt [4]. Inzwischen gibt es empirische Belege für die Wirksamkeit von Ethikberatung in Bezug auf die Zufriedenheit von Angehörigen und Behandelnden, die Unterstützung ethischer Entscheidungsfindung, die wahrgenommenen Auswirkungen auf die klinische Versorgung, die moralische Kompetenz und die Qualität der Kommunikation [2, 10, 22].

Auch in der Psychiatrie verfügen immer mehr Institutionen über *Strukturen der klinischen Ethik*. In der Schweiz ist der Anteil der psychiatrischen Institutionen mit solchen Strukturen im Zeitraum zwischen 2002 und 2020 von gut 10 % auf knapp 60 % angestiegen und liegt damit etwa gleich hoch wie in den somatischen Akutspitälern [28]. Insgesamt am weitesten verbreitet sind Klinische Ethikkomitees (69 %), gut ein Viertel hat klinische Ethiker*innen angestellt und ebenfalls ein Viertel nimmt externe Ethikberatung in Anspruch. In Deutschland bieten laut einer Querschnittserhebung aus dem Jahr 2017 57 % aller psychiatrischen Kliniken klinische Ethikberatung an, wobei die durchschnittliche Anzahl der Beratungen mit 4,1 pro Jahr niedrig ist [26]. In einzelnen Bundesländern wie Nordrhein-Westfalen liegt der Anteil an psychiatrischen Kliniken mit einem Angebot für klinische Ethikberatung deutlich höher [9]. In einer jüngeren Erhebung gaben 66 % der befragten psychiatrischen Einrichtungen an, klinische Ethikberatung anzubieten, wobei der Anteil in forensischen Psychiatrien deutlich geringer war (43 %; [8]).

### Modelle klinischer Ethikberatung

Bis vor 10 Jahren gab es nur wenig *Evaluationsforschung *zu klinischer Ethikberatung in der Psychiatrie [12, 18]. Seither sind einige Publikationen zu vorwiegend qualitativen Studien erschienen, vor allem zur Methode der Moral Case Deliberation (MCD). In einer Längsschnittstudie aus Norwegen entwickelten Gesundheitsfachpersonen, die an mehreren MCD teilgenommen hatten, eine kritischere Haltung gegenüber Interventionen gegen den Willen von Patient*innen, aber Kompetenz, Kooperation oder konstruktive Meinungsverschiedenheiten im Team nahmen insgesamt nicht statistisch signifikant zu [16]. Die Teilnehmenden erlebten MCD überwiegend als positiv, etwa zur Steigerung der moralischen Sensibilität, der Selbstreflexion oder der ethischen Kompetenz [13].

Auch aus Deutschland und der Schweiz liegen Hinweise vor, dass Ethikberatung in der Psychiatrie als hilfreich erlebt wird und zur Konsensfindung beiträgt [14, 20, 27]. In einer Pilotstudie aus der Schweiz nahmen Interventionen gegen den Willen von Patient*innen sowohl in ihrer Häufigkeit als auch in ihrer Intensität nach Implementierung von MCD signifikant ab, was darauf schließen lässt, dass Ethikberatung auch zur Reduktion von Zwang beitragen kann [24]. Mit Ausnahme verschiedener Modelle von MCD sind bisher jedoch kaum Methoden der klinischen Ethikberatung in der Psychiatrie publiziert.

Im vorliegenden Beitrag wird das Basler Modell der prinzipienorientierten klinischen Ethikkonsultation 2.0 vorgestellt, das an der Abteilung Klinische Ethik am Universitätsspital Basel (USB) und an den Universitären Psychiatrischen Kliniken Basel (UPK) entwickelt wurde und dort seit vielen Jahren erfolgreich eingesetzt wird. Anschließend wird auf die besondere Stellung der klinischen Ethik an den UPK eingegangen. Das weiterentwickelte Basler Modell der prinzipienorientierten klinischen Ethikkonsultation 2.0 wird erläutert und dessen Anwendung schrittweise dargestellt.

## Themen der klinischen Ethikkonsultationen an den Universitären Psychiatrischen Kliniken Basel

Die klinischen Ethikkonsultationen an den UPK beziehen sich zu etwa einem Drittel auf die ethische Frage, ob Interventionen gegen den Willen von Patient*innen aufgrund einer Selbst- oder Fremdgefährdung gerechtfertigt sind. Es folgen Fragen zur Nutzen-Schaden-Abwägung von Behandlungen, zur Kindeswohlgefährdung und zum Umgang mit herausforderndem Patientenverhalten (z. B. Aggression, Verwahrlosung, Nichteinhalten der Abteilungsregeln). Weitere Themen der klinischen Ethikkonsultation an den UPK sind unter anderem Fragen im Umgang mit dem Wunsch nach ärztlich assistiertem Suizid, die Begrenzung lebenserhaltender Maßnahmen, der Umgang mit medizinisch nicht indizierten Patientenwünschen, die Versorgungsplanung nach Austritt oder Fragen zur Einwilligungsfähigkeit von Patient*innen (vgl. auch [20]).

Die Abteilung Klinische Ethik bietet neben klassischen Ethikkonsultationen an den UPK auch niederschwellige Beratungsformen wie individuelle Ethikberatungen, teaminterne ethische Fallbesprechungen, Ethikvisiten, Ethikfortbildungen, die Entwicklung von Ethikleitlinien und die Durchführung klinisch-ethischer Projekte an [19].

## Das Basler Modell der prinzipienorientierten klinischen Ethikkonsultation 2.0

**Abb. 1:**
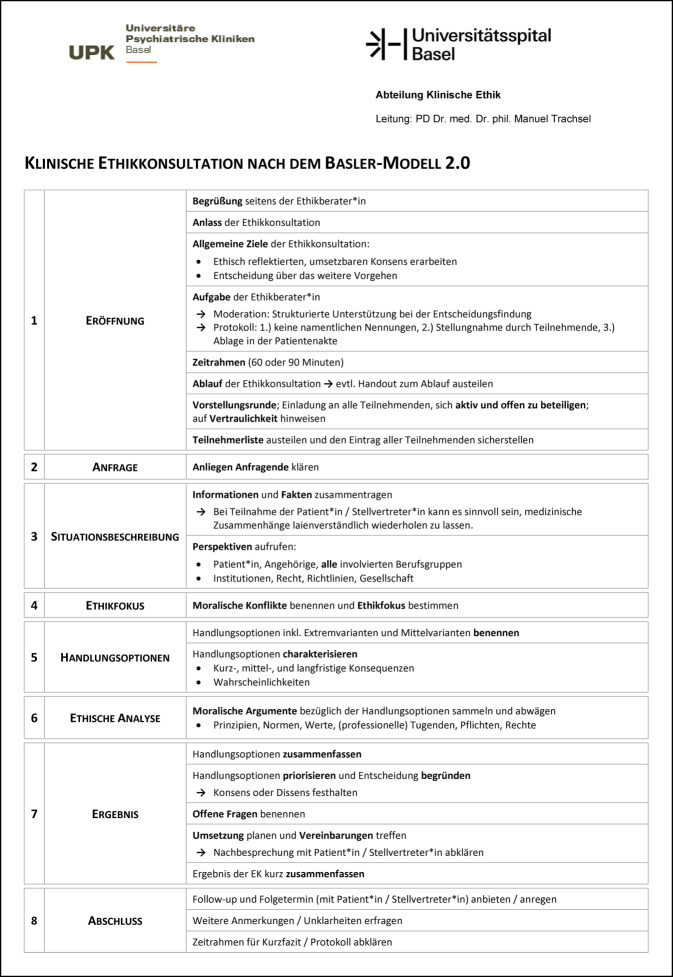
Ablauf einer prinzipienorientierten Ethikkonsultation nach dem Basler Modell 2.0 – Version für Ethikberater*innen. Dieses Dokument kann zur freien Verwendung kostenlos unter folgendem Link heruntergeladen werden: https://www.unispital-basel.ch/medizinische-direktion/klinische-ethik/tools_klinikerinnen

Das Basler Modell der prinzipienorientierten klinischen Ethikkonsultation 2.0 ist eine Weiterentwicklung des Basler Leitfadens zur klinischen Ethikkonsultation [17]. Der Basler Leitfaden orientierte sich formal am „Ethics-facilitation“-Ansatz der American Society for Bioethics and Humanities [1] und normativ am prinzipienethischen Ansatz von Beauchamp und Childress – umgangssprachlich „Vier-Prinzipen-Ansatz“ [3]. Nach dem „Ethics-facilitation“-Ansatz besteht die Rolle der Ethikberater*innen darin, die Entscheidungsträger*innen dabei zu unterstützen, eine Entscheidung zu treffen, die einerseits die Bedürfnisse und Werte der Beteiligten respektiert und sich andererseits im Rahmen der ethischen und rechtlichen Normen bewegt. Ethikberater*innen geben also weder direktiv vor, was das ethisch beste Vorgehen ist, noch begnügen sie sich mit der reinen Vermittlung der vorgefundenen Interessen [1]. Diese grundlegende Orientierung wurde für das Basler Modell der prinzipienorientierten klinischen Ethikkonsultation beibehalten und in ein Vorgehen in acht Schritten überführt (Abb. 1). Für ein weiteres Modell der klinischen Ethikkonsultation, das sich am Vier-Prinzipien-Ansatz orientiert und Parallelen aufweist siehe Marckmann [15].

Das Basler Modell der prinzipienorientierten klinischen Ethikkonsultation 2.0 versteht Ethikkonsultation als ein durch Ethikberater*innen moderiertes Gespräch zur Unterstützung von Behandelnden, Patient*innen oder Angehörigen bei ethischen Problemen in der Patientenversorgung. Alle Mitarbeitenden, Patient*innen oder Angehörige können eine Ethikkonsultation anfragen. An einer Ethikkonsultation nehmen in der Regel zwei Ethikberater*innen (Moderation und Protokoll), die behandelnden Ärzt*innen, Psycholog*innen und Pflegefachpersonen sowie teilweise auch Patient*innen, Angehörige, gesetzliche Betreuer*innen, Vertreter*innen des Rechtsdienstes der Klinik, Therapeut*innen und Sozialarbeiter*innen teil. Im Mittelpunkt der Diskussion steht die Identifikation, Analyse und gemeinsame Bewertung von moralischen Konflikten und Handlungsoptionen mit dem Ziel, ein ethisch gut begründetes und – sekundär – konsensfähiges Vorgehen zu entwickeln. Die Ethikberater*innen sind dabei für die Qualität der ethischen Reflexion verantwortlich, sowohl hinsichtlich des Prozesses der Ethikkonsultation als auch hinsichtlich des Ergebnisses im Sinne der Begründungsqualität der Entscheidung. Die Verantwortung für die Entscheidung und deren konkrete Umsetzung verbleibt jedoch bei den Behandelnden bzw. den Patient*innen. Jede Ethikkonsultation wird von den Ethikberater*innen in einem strukturierten Protokoll dokumentiert, das anschließend in der elektronischen Patientenakte abgelegt wird (Abb. 2).

**Abb. 2:**
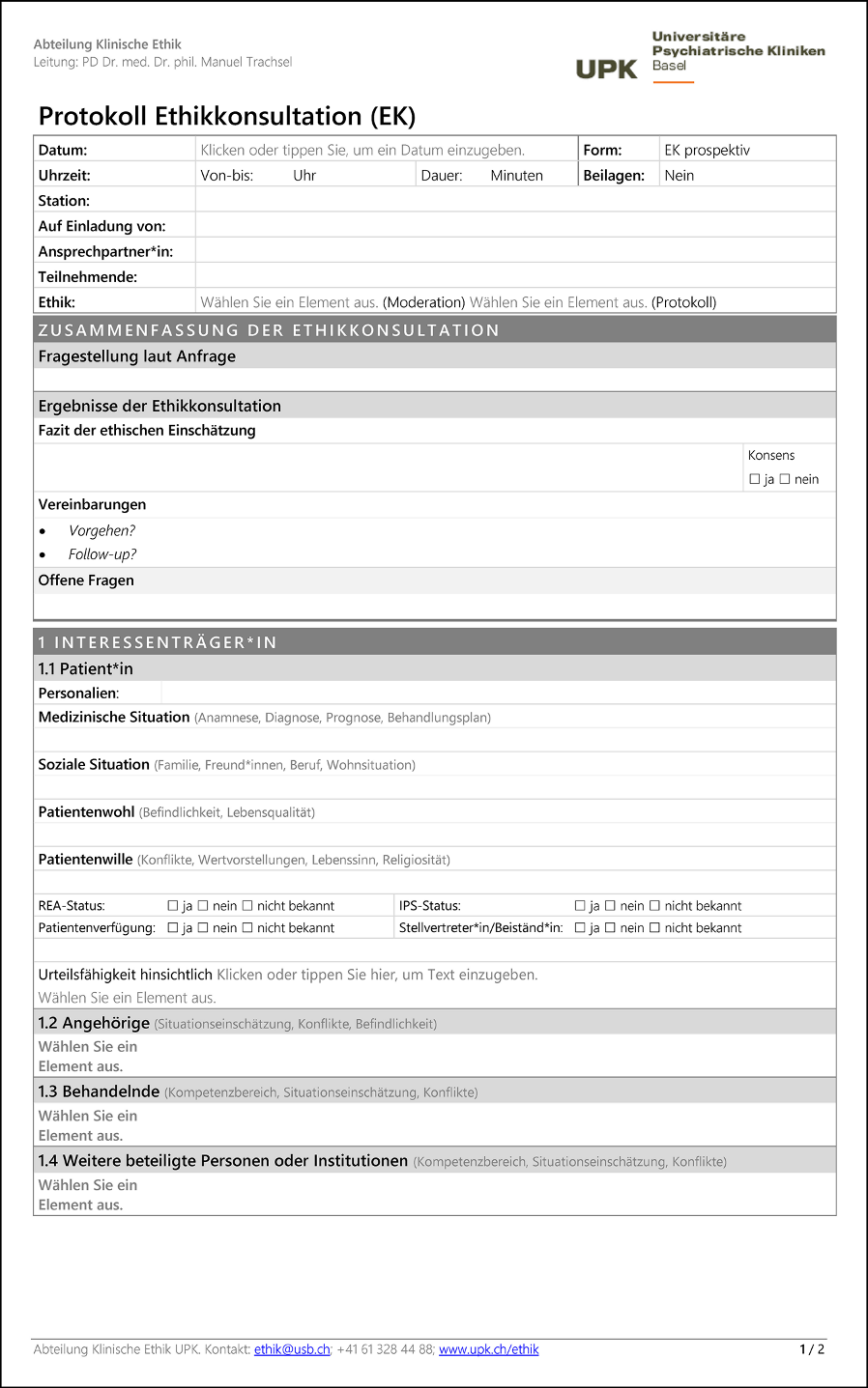
Protokollvorlage zur Dokumentation einer klinischen Ethikkonsultation nach dem Basler Modell 2.0 in den Universitären Psychiatrischen Kliniken (UPK). Eine bearbeitbare Version dieser Vorlage kann zur freien Verwendung kostenlos unter folgendem Link heruntergeladen werden: https://www.unispital-basel.ch/medizinische-direktion/klinische-ethik/tools_klinikerinnen. *IPS* Intensivpflegestation, *REA* Reanimation

**Abb. 2:**
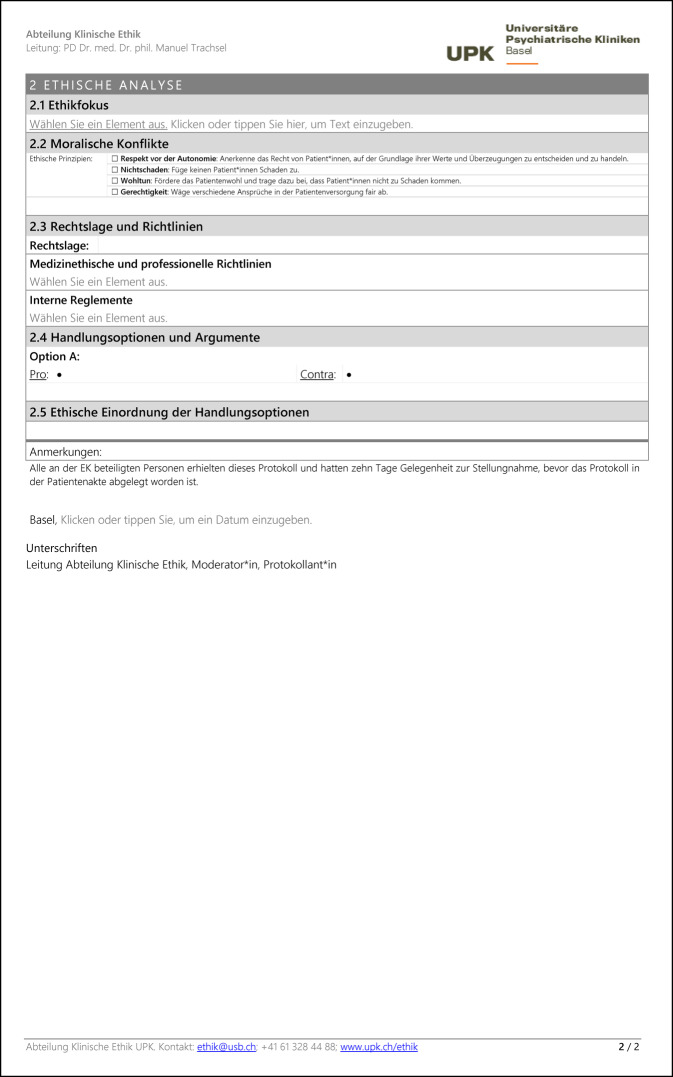
(Fortsetzung)

## Schritte der prinzipienorientierten Ethikkonsultation nach dem Basler Modell 2.0

### Eröffnung

Die *Eröffnung der Ethikkonsultation* ist zentral, um alle Teilnehmenden über die Modalitäten zu informieren und um eine vertrauensvolle Atmosphäre zu schaffen, in der sich alle Teilnehmenden offen und konstruktiv einbringen können.

**Abb. 3:**
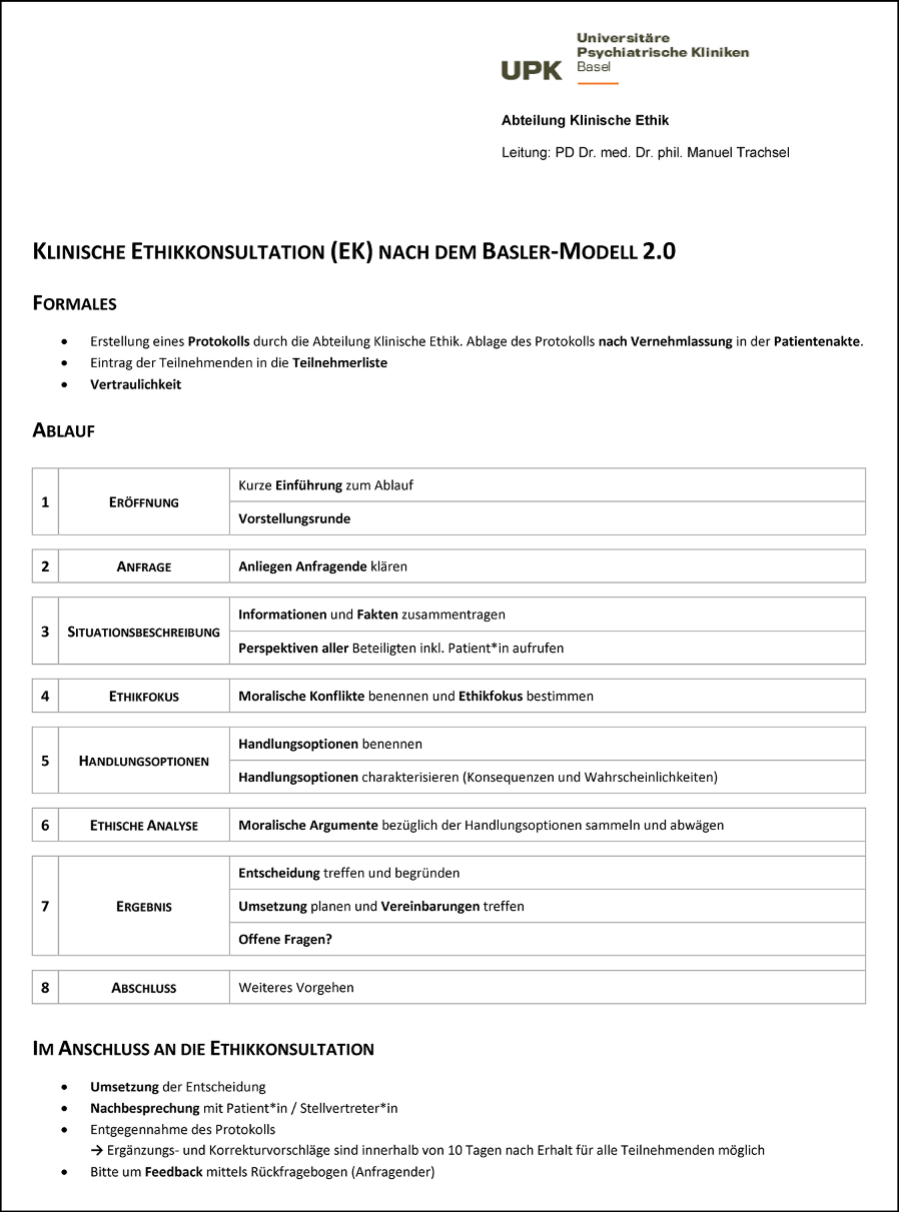
Handout für Teilnehmende zum Ablauf einer prinzipienorientierten Ethikkonsultation nach dem Basler Modell 2.0. Dieses Handout kann zur freien Verwendung kostenlos unter folgendem Link heruntergeladen werden: https://www.unispital-basel.ch/medizinische-direktion/klinische-ethik/tools_klinikerinnen

Nach der Begrüßung der Teilnehmenden rekapituliert der*die moderierende Ethikberater*in den Anlass der Ethikkonsultation. Dazu gehört eine Beschreibung der allgemeinen Ziele einer Ethikkonsultation und der Aufgaben der Ethikberater*innen hinsichtlich Moderation und Protokoll. Anschließend wird der zeitliche Rahmen geklärt und der Ablauf der prinzipienorientierten Ethikkonsultation nach dem Basler Modell 2.0 in acht Schritten skizziert. Damit die Teilnehmenden während der Ethikkonsultation den Überblick über die Schritte behalten, wird der Ablauf zusätzlich auf einem Handout verteilt (Abb. 3). Anschließend folgt eine Vorstellungsrunde.

### Anliegen

Die anfragende Person wird eingangs gebeten, den Anlass und ihr *Anliegen an die Ethikkonsultation* darzulegen. Dabei geht es noch nicht um eine detaillierte Situationsbeschreibung, die im nächsten Schritt erfolgt, sondern um eine Darstellung des moralischen Problems aus Sicht und in den Worten der anfragenden Person sowie ihre Erwartungen an die Ethikkonsultation. Dieser Schritt dient dazu, die moralische Ausgangslage der Ethikkonsultation zu verstehen, die Zielsetzung der Ethikkonsultation zu spezifizieren und bietet den übrigen Teilnehmenden die Möglichkeit, andere Anliegen an die Ethikkonsultation zu formulieren.

### Situationsbeschreibung

Es folgt eine möglichst genaue *Sammlung aller Informationen*, die für eine ethische Analyse der Situation relevant sind. Dazu gehören insbesondere der Gesundheitszustand, die Prognose und die aktuelle Versorgungssituation, der aktuelle, mutmaßliche oder vorausverfügte Wille des Patienten oder der Patientin, die bisherigen Lebensumstände, die Lebensziele und das soziale Umfeld des Patienten oder der Patientin sowie die strukturellen, medizinethischen und rechtlichen Voraussetzungen der Behandlung. Die Ethikberater*innen achten dabei darauf, dass die Perspektiven aller Stakeholder – auch derjenigen, die nicht an der Ethikkonsultation teilnehmen – eingeholt und allfällige Wissenslücken identifiziert werden. Bei der Teilnahme von Patient*innen oder Stellvertreter*innen ist darauf zu achten, dass medizinische Zusammenhänge laienverständlich erklärt werden.

### Ethikfokus

Nach Abschluss der Informationssammlung werden die bestehenden *moralischen Grundkonflikte* benannt und der sogenannte *Ethikfokus* festgelegt. Die moralischen Grundkonflikte zu identifizieren und zu artikulieren ist eine Aufgabe, die normalerweise der*die Moderator*in übernimmt. Nach dem Basler Modell werden moralische Grundkonflikte, wenn möglich, als Konflikte zwischen zwei oder mehr Prinzipien des Vier-Prinzipien-Ansatzes dargestellt. Dabei kann es hilfreich sein, die vier Prinzipien kurz zu charakterisieren. Da in einer Situation mehrere moralische Konflikte auftreten können, wird anschließend in Absprache mit den Beteiligten der Ethikfokus bestimmt, d. h. der moralische Hauptkonflikt, der einem Fortschritt in der Situation unmittelbar entgegensteht und der in der Ethikkonsultation weiter behandelt werden soll. Dieser Ethikfokus kann sich formal und inhaltlich von der ursprünglichen Fragestellung unterscheiden. Dieser Schritt umfasst noch keine detaillierte ethische Analyse, sondern dient dazu, eine gemeinsame Orientierung über die moralische Problemlage zu gewinnen und die Diskussion auf einen Hauptkonflikt zu fokussieren.

### Handlungsoptionen

Nach der Bestimmung des Ethikfokus werden die *Handlungsoptionen* benannt, die in der aktuellen Situation zur Verfügung stehen. Diese können medizinische, aber auch soziale, rechtliche oder administrative Interventionen (z. B. Einbezug einer Vertrauensperson, Information von Behörden oder Verlegung des*der Patient*in) umfassen. Unkonventionelle Handlungsoptionen können ebenfalls in Betracht gezogen werden, jedoch muss die Aussicht bestehen, dass diese Handlungsoptionen mit den zur Verfügung stehenden Mitteln realisiert werden können. Dabei hat sich die (grafische) Darstellung auf einem Spektrum von Handlungsoptionen bewährt, d. h. von Optionen, die eindeutig einen Pol des moralischen Grundkonflikts favorisieren, über Optionen, die einen Ausgleich zwischen den Polen suchen, bis hin zu Optionen, die eindeutig den anderen Pol des moralischen Grundkonflikts favorisieren. Die zur Verfügung stehenden Handlungsoptionen werden hinsichtlich ihrer kurz-, mittel- und langfristigen Folgen sowie ihrer Eintrittswahrscheinlichkeit möglichst genau beschrieben, wobei Prognoseunsicherheiten explizit zu berücksichtigen sind. Eine Bewertung der Handlungsoptionen ist damit noch nicht verbunden – diese erfolgt erst in den nächsten beiden Schritten.

### Ethische Analyse

Die *ethische Analyse* erfolgt für jede Handlungsoption einzeln, indem moralische Argumente für oder gegen die Handlungsoptionen gesammelt werden. Mit moralischen Argumenten sind hier umgangssprachlich normative Aussagen („Gründe“) gemeint, die eines der vier ethischen Prinzipien oder auch andere Normen, Tugenden oder Pflichten zum Ausdruck bringen und aus denen sich zusammen mit deskriptiven Aussagen praktische Handlungsempfehlungen ableiten lassen (z. B. „Einwilligungsfähige Patient*innen sollten psychiatrische Interventionen ablehnen dürfen“ oder „Behandelnde sollten eine tragfähige therapeutische Beziehung wahren“). Auch hier kann es hilfreich sein, wenn der*die Ethikberater*in die Handlungsoptionen vor dem Hintergrund der vier ethischen Prinzipien betrachtet und entsprechend prinzipienbasierte Argumente ergänzt. Es kann auch erforderlich sein, dass der*die Ethikberater*in eine begriffliche Klärung oder eine medizinethische Einschätzung vornimmt (z. B. Voraussetzungen der Einwilligungsfähigkeit oder Gültigkeit einer Patientenverfügung).

Wenn die prinzipienbasierten Argumente für alle Handlungsoptionen zusammengetragen wurden und sich aus ethischer Sicht keine eindeutig zu favorisierende Handlungsoption abzeichnet, empfiehlt es sich, gemeinsam mit den Teilnehmenden eine explizite Gewichtung der Argumente vorzunehmen. Dazu kann es hilfreich sein, den Argumenten einen Wert auf einer einfachen Plus-Minus-Skala zuzuordnen (++ = sehr wichtiges Proargument, + = wichtiges Proargument, Ø = kein wichtiges Argument, – = wichtiges Kontraargument, – – = sehr wichtiges Kontraargument), was einen Überblick über die argumentative Stärke der jeweiligen Handlungsoptionen ermöglicht.

### Ergebnis: Ethische Synthese

In diesem Schritt werden die Handlungsoptionen mit den jeweiligen prinzipienbasierten Argumenten und deren Gewichtung durch den*die Moderator*in zusammengefasst. Anschließend stellt er*sie die *Priorisierung der Handlungsoptionen* entsprechend der gemeinsamen Gewichtung vor und fragt die Teilnehmenden, ob sie mit dieser Priorisierung einverstanden sind. Der Konsens bzw. Dissens wird explizit festgehalten. Ein vernünftiger Dissens zwischen ethisch vergleichbar gut begründeten Positionen ist in der prinzipienorientierten Ethikkonsultation nach dem Basler Modell 2.0 durchaus möglich, auch wenn ein solcher in unserer Beratungspraxis nicht häufig vorkommt. Die Entscheidungsverantwortung bleibt bei den Teilnehmenden und jede*r muss sein*ihr Handeln letztlich selbst moralisch verantworten – die klinische Ethikkonsultation bleibt explizit beratend.

Im Anschluss werden *offen gebliebene Fragen* durch den*die Moderator*in festgehalten und die Umsetzung der Handlungsoption durch konkrete *Vereinbarungen* zwischen den Teilnehmenden geplant. Im Fall eines Dissenses wird festgehalten, auf welche Schritte man sich einigen kann und ob bzw. welche weiteren Schritte zur Klärung unternommen werden sollen. Ebenso ist zu klären, wer das Ergebnis der klinischen Ethikkonsultation mit dem*der Patient*in bespricht – in der Regel geschieht dies im Gespräch mit dem Behandlungsteam, wobei die Ethikberater*innen auf Wunsch am Gespräch teilnehmen. Wir empfehlen eine Zusammenfassung des Ergebnisses inklusive der getroffenen Vereinbarungen durch den*die Moderator*in.

### Abschluss

Abschließend werden letzte *organisatorische Fragen* geklärt, etwa ob und wann eine ethische Folgekonsultation stattfinden soll, ob bezüglich der Umsetzung noch Unklarheiten bestehen oder wann das schriftliche Fazit oder das Protokoll der Ethikkonsultation zur Verfügung steht. Allen Teilnehmenden wird für die Teilnahme an der Ethikkonsultation herzlich gedankt.

## Schlussbemerkungen

In der Psychiatrie ergeben sich im Vergleich zur somatischen Medizin spezifische ethische Herausforderungen, die im Rahmen der Psychiatrie- und Psychotherapieethik zunehmend untersucht werden. Entscheide in psychiatrischen Behandlungssettings haben oft einen längeren Zeithorizont und einen breiteren sozialen Horizont als in der somatischen Medizin. Diese Charakteristika psychiatrischer Entscheidungsfindung wirken sich auch auf den Bedarf, die Strukturen und den Ablauf klinischer Ethikkonsultation aus [19]. Studien zu den spezifischen Anforderungen der klinischen Ethikberatung in der Psychiatrie gibt es jedoch noch kaum.

Das generische Basler Modell der prinzipienorientierten klinischen Ethikkonsultation 2.0 ist nach unserer Einschätzung auch für den psychiatrischen Kontext gut geeignet. Erste Auswertungen deuten darauf hin, dass Ethikkonsultationen nach dem Basler Modell geeignet sind, in schwierigen Behandlungssituationen umsetzbare Lösungen zu finden, einen Konsens zwischen den verschiedenen Akteuren herzustellen und dass die Ethikkonsultationen von den Behandelnden als hilfreich erlebt werden [20]. Der Vier-Prinzipien-Ansatz der biomedizinischen Ethik kann die Struktur ethischer Konflikte auch in der psychiatrischen Patientenversorgung adäquat abbilden. Das klar strukturierte Vorgehen des Modells ermöglicht es, auch komplexe Entscheidungssituationen übersichtlich darzustellen, einen klaren Ethikfokus festzulegen und zu einer sorgfältigen ethischen Abwägung der Handlungsoptionen zu gelangen. Eine systematische Evaluation der prinzipienorientierten klinischen Ethikkonsultation nach dem des Basler Modell 2.0 steht allerdings noch aus und soll zukünftig durchgeführt werden.

Im Rahmen von Ethikkonsultationen in der Psychiatrie sind im Einzelfall neben den Besonderheiten der psychiatrischen Diagnostik, Prognostik und Therapie immer auch spezifische psychiatrieethische oder psychiatrierechtliche Fragen zu diskutieren, etwa zur Beurteilung der Einwilligungsfähigkeit, zu psychiatrischen Patientenverfügungen, zum Umgang mit Suizidalität, zur Offenlegung von Informationen bei Fremdgefährdung oder zur legitimen Anwendung von Zwang in der Behandlung psychisch erkrankter Personen. Dies erfordert von den Ethikberater*innen spezifische Kenntnisse im Bereich der Psychiatrie, der psychiatrischen Ethik und des psychiatrischen Rechts.

Neben der einzelfallbezogenen Ethikkonsultation haben sich zudem in der Psychiatrie auch andere Praxisformen der ethischen Unterstützung bewährt, etwa die individuelle Ethikberatung von Einzelpersonen, die Unterstützung bei der Entwicklung ethisch relevanter Richtlinien oder die ethische Fort- und Weiterbildung zur Förderung der ethischen Kompetenz der Gesundheitsfachpersonen.

## Fazit für die Praxis

- In der Psychiatrie und Psychotherapie sind Fachpersonen mit einem breiten Spektrum ethischer Herausforderungen konfrontiert.
- Zur Unterstützung bei der Identifikation und Formulierung moralischer Konflikte und zu deren Bearbeitung und Lösungsfindung hat es sich bewährt, dass klinisch tätige Fachpersonen niederschwellig klinische Ethikberatung durch professionelle Ethikberater*innen anfragen können.
- Die Wirksamkeit klinischer Ethikberatung ist empirisch mittlerweile gut validiert. Es braucht jedoch weitere wissenschaftliche Studien zum Nutzen und zur differenziellen Indikation der verschiedenen Formen und Modelle klinischer Ethikberatung.
- Klinische Ethikberatung etabliert sich zunehmend in psychiatrischen Institutionen.
- Die prinzipienorientierte klinische Ethikkonsultation nach dem Basler Modell 2.0 bietet ein schrittweises Vorgehen zur Bearbeitung ethischer Fragen, das sich auch im psychiatrischen Setting bewährt hat.
